# Total and cause-specific mortality in patients with personality disorders: the association between comorbid severe mental illness and substance use disorders

**DOI:** 10.1007/s00127-021-02055-3

**Published:** 2021-03-07

**Authors:** Anne Høye, Bjarne K. Jacobsen, Jørgen G. Bramness, Ragnar Nesvåg, Ted Reichborn-Kjennerud, Ina Heiberg

**Affiliations:** 1 Department of Clinical Medicine, UiT—The Arctic University of Norway, pb 6124, 9291 Tromsø, Norway; 2 Division of Mental Health and Substance Abuse, University Hospital of North Norway, Tromsø, Norway; 3 Center for Clinical Documentation and Evaluation (SKDE), Tromsø, Norway; 4 Department of Community Medicine, UiT—The Arctic University of Norway, Tromsø, Norway; 5 Centre for Sami Health Research, UiT—The Arctic University of Norway, Tromsø, Norway; 6 Norwegian Institute of Public Health, Oslo, Norway; 7 Norwegian National Advisory Unit on Concurrent Substance Abuse and Mental Health Disorders, Hamar, Norway; 8 Norwegian Medical Association, Oslo, Norway; 9 Institute of Clinical Medicine, University of Oslo, Oslo, Norway

**Keywords:** Mortality, Personality disorders, Comorbidity, Substance use disorders, Severe mental illness

## Abstract

**Purpose:**

To investigate the mortality in both in- and outpatients with personality disorders (PD), and to explore the association between mortality and comorbid substance use disorder (SUD) or severe mental illness (SMI).

**Methods:**

All residents admitted to Norwegian in- and outpatient specialist health care services during 2009–2015 with a PD diagnosis were included. Standardized mortality ratios (SMRs) with 95% confidence intervals (CI) were estimated in patients with PD only and in patients with PD and comorbid SMI or SUD. Cox proportional hazards models were used to estimate adjusted hazard ratios (HRs) with 95% CIs in patients with PD and comorbid SMI or SUD compared to patients with PD only.

**Results:**

Mortality was increased in both in- and outpatients with PD. The overall SMR was 3.8 (95% CI 3.6–4.0). The highest SMR was estimated for unnatural causes of death (11.0, 95% CI 10.0–12.0), but increased also for natural causes of death (2.2, 95% CI 2.0–2.5). Comorbidity was associated with higher SMRs, particularly due to poisoning and suicide. Patients with comorbid PD & SUD had almost four times higher all-cause mortality HR than patients with PD only; young women had the highest HR.

**Conclusion:**

The SMR was high in both in- and outpatients with PD, and particularly high in patients with comorbid PD & SUD. Young female patients with PD & SUD were at highest risk. The higher mortality in patients with PD cannot, however, fully be accounted for by comorbidity.

**Supplementary Information:**

The online version contains supplementary material available at 10.1007/s00127-021-02055-3.

## Introduction

Personality disorders (PDs) are characterized by personality traits causing suffering, reduced quality of life and often reduced ability to maintain interpersonal relationships [1, 2]. PDs are etiologically complex diagnostic categories with a high prevalence of comorbidity with other mental disorders in both population-based [2–6] and clinical samples [7]. A meta-analysis from 2020 including studies based on both DSM and ICD criteria shows the worldwide pooled prevalence of PDs in the general population to be 7.8%, and higher in high-income countries (9.6%)[8]. Patients with PDs are in frequent and in some cases long-term contact with health care services due to their mental illness. Studies have demonstrated that 24%of patients in primary care meet criteria for a PD diagnosis [9], and 50% of all patients in psychiatric outpatient care [10]. The prevalence rate among psychiatric inpatients is shown to be considerably lower; about 15% for PD as primary or secondary diagnosis [11]. The prevalence is higher for women than for men in specialist health care services, while the opposite is the case in community settings [2, 10, 12].

Approximately one in five people with bipolar disorder is also diagnosed with borderline or emotionally unstable PD [13], affective and anxiety disorders are strongly associated with schizotypal PD [14], and the prevalence rate of PDs in individuals at high risk for psychosis has been found to be as high as 39% [15]. There is also a well-known relationship between PDs and substance use disorder (SUD) [16–24]. A clinical study from Denmark found comorbid SUD in 39% of patients with PDs [16], and a systematic review from 2000 estimates that 38–57% of patients with borderline PD have at least one SUD diagnosis [24]. The co-occurrence of PDs with other mental disorders or SUD in the same individual can be explained by a number of factors [25] including common genetic vulnerability [26–29], shared environmental factors such as trauma and abuse exposure [20, 30, 31], externalizing/internalizing dimensions, and common liability factors in different disorders [32–35]. In a population based, Norwegian twin study, genetic risks for borderline and antisocial PD explained 32–60% of the total variance in cannabis use and cannabis use disorder [26, 27]. Genetic risk for dependent PD explained 11% of the variance, whereas genetic risk for avoidant PDs explained 16% of the variance.

In a study by Nordentoft and coworkers, the highest observed/expected mortality ratios (standardized mortality ratios, SMRs) were found for SUD and PD [36]. Other studies show a similar pattern [37–42], also in patients receiving only outpatient care [43]. The increased mortality may to a large degree be explained by a higher prevalence of unnatural causes of death such as suicide and accidents, especially in borderline PD [41, 44–48], but also a higher prevalence of natural causes of death [12, 39, 42, 43, 49, 50].

Even though the co-occurrence of PDs and other mental disorders is shown to be associated with the course and treatment outcome negatively [1, 2, 4], very few studies have addressed the association between psychiatric comorbidity and mortality in individuals with PD. Björkenstam et al. [42] reported SMRs in patients with PD to be 3.3 for women and 2.4 for men, whereas SMR in patients who had also been admitted with other psychiatric or somatic diseases was 6.5 for women and 5.7 for men. In 2018, Kuo and coworkers were the first to publish data on the association between comorbid SUD and other axis I psychiatric disorders on SMRs among patients with a PD admitted to hospital [12]. SMRs for all-cause mortality of PD only, PD with non-SUD mental disorders and PD with SUD were 4.5, 7.4, and 16.0, respectively. Kjær and coworkers found SMR of patients with borderline PD to be 8.3 [47], and that more than three yearly hospital admissions or a comorbid SUD were factors significantly associated with higher mortality. To our knowledge, no previous study has investigated the association between comorbid SUD or SMI and mortality in patients with PDs admitted to outpatient services.

The aims of the present study were to use prospective data from nationwide health registries to estimate the mortality (all-cause mortality as well as mortality due to natural and unnatural deaths) in both in- and outpatients with PD compared with the general population, and to explore the impact of comorbid SUD or SMI (defined as schizophrenia, bipolar disorder or major depression), or a combination of these disorders, on mortality in patients with PDs.

## Materials and methods

### Study population

All patients aged 20–79 years who received a diagnosis of PD in the Norwegian Patient Registry (NPR) between 2009 and 2015 were included. NPR contains information about all in- and outpatient treatment in somatic and psychiatric specialized health care in Norway (i.e., state-owned hospitals and outpatient clinics, government-funded substance use treatment facilities, and private health clinics with governmental reimbursement). The NPR has almost complete coverage from 2009. Patients were excluded if a valid social security number was missing (*n* = 2186), a diagnosis of intellectual disability (*n* = 365) or inconsistent data regarding age, gender or emigration date (*n* = 27) were recorded.

### Diagnostic categories

Diagnostic codes in the NPR follow the International Classification of Diseases and Related Health Problems, 10th Revision (ICD-10) [51]. Patients were included in the PD group if they had at least one ICD-10 code F60 recorded in the NPR during 2009–2015. Patients identified exclusively in somatic care constituted 2% of the PD group.

Comorbid SMI was considered present if a diagnosis of schizophrenia-related disorder (codes F20-F29), bipolar disorder (codes F30-F31) or major depression (codes F32.2-F32.3, F33.2-F33.3) was recorded from 2009 to 2015. Comorbid SUD was considered present if a diagnosis of mental and behavioral disorders due to use of psychoactive substances (F10-F19, excluding tobacco (F17)), or a somatic diagnosis strongly indicating alcohol abuse (codes K70, E52, E24.4, G31.2, G62.1, G72.1, I42.6, K29.2, K86.0, O35.4, Y57.3, Z50.2, Z50.3, Z71.4, and Z72.1) was recorded between 2009 and 2015. Patients identified by somatic diagnoses only constituted 0.5% of the SUD group. In addition, we included in the PD & SUD group 393 PD patients with repeated episodes in drug treatment facilities without any registered diagnosis of SUD. This group was considered to be patients with unknown SUD.

### Information on causes of death

The unique social security number assigned to all Norwegian residents was used to link information from the NPR to two other nationwide population-based registries; the National Population Registry and the Cause of Death Registry. The National Population Registry provided information on the year of birth, emigration and date of death. The Cause of Death Registry, which includes information about 98% of all deaths in Norway [52], provided data on the underlying cause of death. Causes of death were based on death certificates coded by the physician who examined the deceased. Overall, approximately 8% of cases in the Cause of Death Registry were supplemented by autopsy data. Causes of death were coded according to ICD-10, and divided into natural causes (A00-R99), unnatural causes (V01-Y98), and missing cause of death. Natural causes of death were further divided into cancer (C00-C97), cardiovascular (I00-I99), respiratory (J00-J99), mental and behavioral (F00-F99), and other natural causes. Unnatural causes of death were divided into poisoning (X40-X49), suicide (X60-X84 and Y87.0), and other unnatural causes. The reference population included all residents in Norway who for each of the years 2009 to 2015 were between 20 and 79 years of age by the end of the year. Annual number of deaths in sex-stratified five-year age groups for the reference population were obtained from the Norwegian Institute of Public Health [53], and annual population figures in the age groups 20–79 in the years 2009–2015 were obtained from Statistics Norway [54].

### Statistical analysis

To study the association between psychiatric comorbidity on relative mortality, we split the PD group into three groups; PD only (e.g., no diagnosis of SMI or SUD in the period), PD & SMI (without SUD), and PD & SUD (with or without SMI). We merged the two groups of PD & SUD with and without SMI in the analyses, but sensitivity analyses were performed. We calculated the relative mortality compared to the general population (SMRs) with corresponding 95% confidence intervals (CIs) for all patients with PD, patients with PD only, PD & SMI (but not SUD), and PD & SUD (with and without SMI). To examine the impact of diagnostic instability or uncertainty, we performed sensitivity analyses including (i) only individuals with a single PD diagnosis and (ii) only individuals with repeated PD diagnosis.

The SMR is computed as the observed number of deaths divided by the expected number of deaths, and shows the relative mortality of the patient group compared to that of the general population of similar age and sex. The expected number of deaths was calculated as the total number of person-years at risk in each sex-, age group-, and calendar year band, multiplied by the corresponding age- (5-year age groups), sex-, and calendar-year specific (2009–2015) death rate in the general population. Age was defined as attained age at the end of each calendar year. Person-years at risk contributed by persons who moved from one age band to the next during follow-up was assigned to the respective sex-, age group-, and calendar year bands, using the “lexis” method [55]. The study cohort was followed from the date of their first consultation or admission with a PD diagnosis in the period 2009–2015. Patients already hospitalized before January 1, 2009 (*n* = 312) were followed from this date. Follow-up ended on December 31, 2015, on the date of emigration, on December 31 in the year of the 79th birthday, or on the date of death, whichever came first. We present sex-specific SMRs for all PD patients according to cause of death, followed by SMRs for PD only, PD & SMI and PD & SUD, overall and stratified by sex and in- or outpatient status.

To compare mortality within the PD group, we used Cox proportional hazards models with attained age as the underlying time scale to estimate adjusted hazard ratios (HRs) with 95% CIs in patients with PD and comorbid SMI or SUD compared to patients with PD only. In patients with PD & SUD, the association was clearly attenuated by attained age (stronger relationship in younger patients). Thus, we also present analyses stratified according to age group (age at start of follow-up 15–34 years, 35–49 years and 50–79 years). The analyses were performed using SAS statistical software, version 9.4 (SAS Institute Inc., Cary, N.C.).

## Results

### Characteristics of the study populations

We identified 32,885 adult patients with PD (60% females) (Table 1). A majority of the patients (70%) were treated as outpatients only; 86% in PD only, 59% in PD & SMI, and 48% in PD & SUD, respectively. The most common PDs were F60.3 Emotionally unstable PD (30.3%), F60.6 Anxious (avoidant) PD (27.9%), and F60.9 Unspecified PD. The patients were followed for a mean of 4.0 years (SD 2.3). Women were overrepresented in the PD only group (63%) and in the PD & SMI group (66%). Mean age at entry was 37.4 years (SD 12.0) in patients with PD only, 38.8 (SD 12.5) in patients with PD & SMI, and 36.2 (SD 11.7) in patients with PD & SUD.

**Table 1:** Demographic and clinical characteristics of the study population

	All PD patients		PD only		PD & SMI		PD & SUD^a^	
*Patients, n*	32,885		17,432		5833		9620	
Women, n (%)	19,747	(60.0)	11,000	(63.1)	3830	(65.7)	4,917	(51.1)
*Age at entry, mean (SD)*	37.3	(12.0)	37.4	(12.0)	38.8	(12.5)	36.2	(11.7)
Women	36.8	(12.3)	36.8	(12.8)	38.2	(12.8)	35.8	(12.3)
Men	38.0	(11.6)	38.5	(12.0)	40.0	(12.0)	36.6	(11.0)
*Treatment level, n (%)*								
Inpatient treatment	9,858	(30.0)	2,481	(14.2)	2,389	(41.0)	4,988	(51.9)
Outpatient treatment only	23,027	(70.0)	14,951	(85.8)	3,444	(59.0)	4,632	(48.1)
*SUD, n (%)*								
Alcohol use disorder only^b^	2298	(7.0)	0		0		2298	(23.9)
Cannabis use disorder only	555	(1.7)	0		0		555	(5.8)
Hard drug use disorders only^c^	1121	(3.4)	0		0		1121	(11.7)
Mixed drug use disorder^d^	5253	(16.0)	0		0		5253	(54.6)
Unknown drug use disorder^e^	393	(1.2)	0		0		393	(4.1)
*Severe mental disorders, n (%)*								
Schizophrenia	2860	(8.7)	0		1520	(26.1)	1340	(13.9)
Bipolar disorder	3956	(12.0)	0		2451	(42.0)	1505	(15.6)
Major depression	4261	(13.0)	0		2717	(46.6)	1544	(16.0)
Deaths	890	(2.7)	248	(1.4)	181	(3.1)	461	(4.8)
Women, *n* (%)	434	(1.3)	138	(0.8)	103	(1.8)	193	(2.0)
Total follow-up (person-years)	131,042		68,399		24,266		38,377	
Follow up per patient, person-years, mean (SD)	4.0	(2.3)	3.9	(2.3)	4.2	(2.2)	4.0	(2.2)
Deaths per 1000 person-years	6.8		3.6		7.5		12.0	

*PD* personality disorder, *SMI* severe mental illness, *SUD* substance use disorder, *n* number of patients, *SD* standard deviation

^a^Includes patients with PD & SUD, with and without SMI

^b^ICD-10 codes F10, K70, E52, E24.4, G31.2, G62.1, G72.1, I42.6, K29.2, K86.0, O35.4, O35.5, Y57.3, Z50.2, Z71.4, and Z72.1

^c^ICD-10 codes F11, F13, F14, F15, F16, and Z50.3

^d^ICD-10 code F19 or combinations of other F1-diagnoses

^e^Includes persons with repeated episodes at substance use treatment facilities, but no diagnosis of SUD or pathological gambling

Rates of psychiatric comorbidity were high. With categories not mutually exclusive, the most common comorbid diagnosis among those with PD & SMI was major depression (47%), whereas 42% had a registered bipolar disorder diagnosis and 26% a schizophrenia-related diagnosis. In the PD & SUD group, the majority of the patients (55%) had registered mixed drugs use disorder (ICD-10 code F19 Mental and behavioral disorders due to multiple drug use and use of other psychoactive substances), or a combination of other F1-diagnoses. 24% had alcohol use disorder only, 12% had hard drug use only (ICD-10 codes F11, F13, F16, and Z50.3), 6% had cannabis use disorder only, and 4% had unknown drug use disorder.

### All-cause and cause-specific mortality

**Fig. 1:**
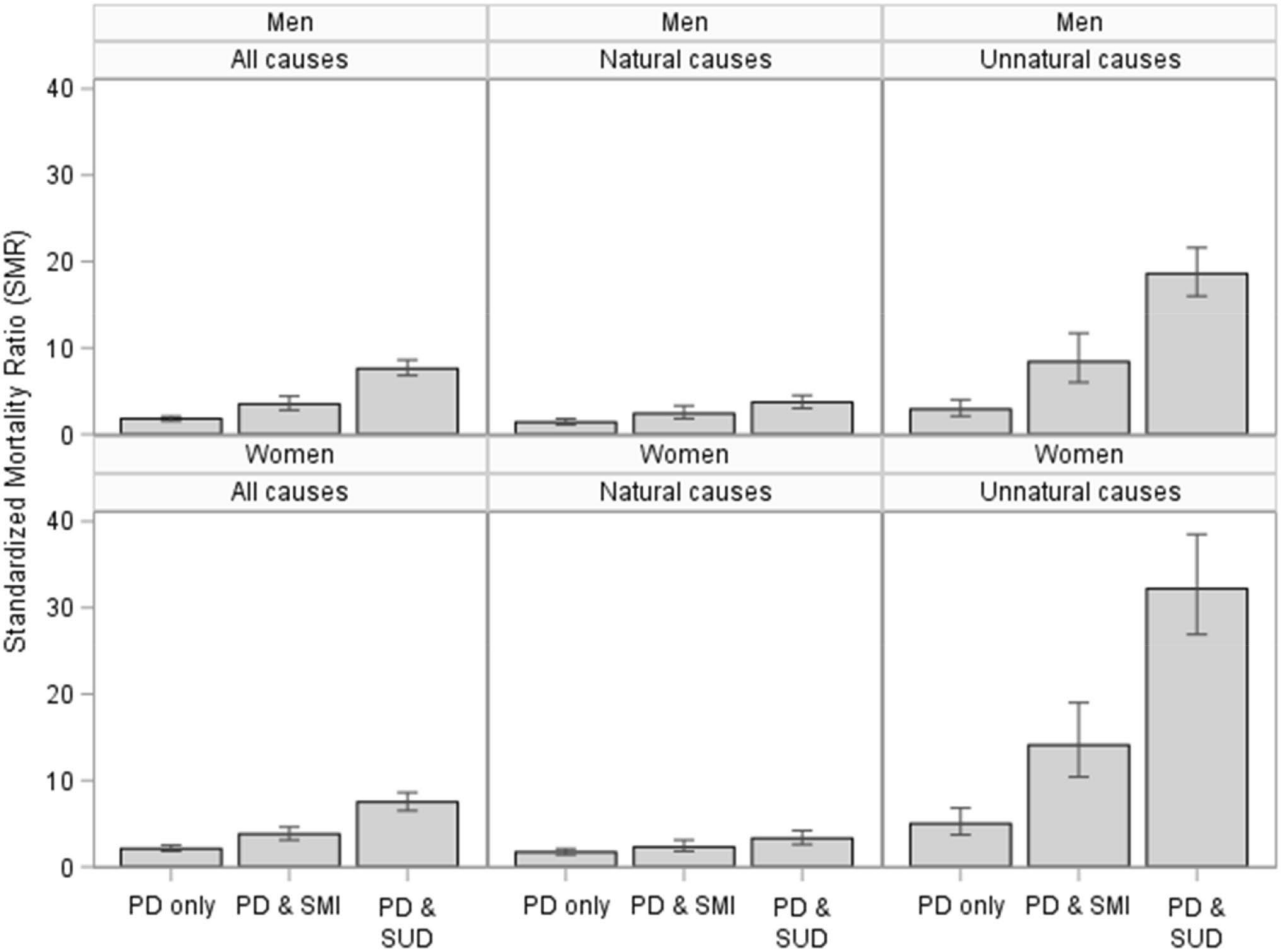
Sex-specific all-cause and cause-specific standardized mortality ratios with 95% confidence intervals for patients with personality disorder with and without psychiatric comorbidity

Of the 32,885 patients included, 890 died during the follow-up, 434 women (2.2%) and 456 men (3.5%). Mortality due to both natural and unnatural deaths was elevated in both PD only, PD & SMI, and PD & SUD, compared to what was expected in the general population. As shown in Table 2, comorbidity was generally related to higher SMRs. There were overall significantly higher SMRs in patient with PD & SMI (but no SUD) compared to patients with PD only, and significantly higher SMRs in patients with PD & SUD (with and without SMI) compared to patients with PD & SMI without SUD. The largest association between comorbidity and SMRs was observed for unnatural causes in both men and women, as displayed in Fig. 1. Comorbidity was associated with particularly high SMRs due to unnatural causes, 3.8 (95% CI 3.0–4.7) in PD only, 11.1 (95% CI 8.9–13.8) in PD & SMI, and 22.5 (95% CI 20.1–25.3) in PD & SUD. In the PD & SUD group, SMR was extremely high for poisoning (36.7, 95% CI 30.9–43.7) but also for suicide (23.9, 95% CI 20.1–28.5). The mortality in patients with PD & SUD was higher also due to natural causes of death; death because of respiratory diseases having a ninefold and cardiovascular death a twofold increase compared to the general population. For natural causes of death in patients with comorbid PD & SMI, SMR was also highest for respiratory diseases, albeit substantially lower than in patients with PD & SUD. Mortality was higher for natural and unnatural causes of death in both inpatients and patients treated solely in outpatient settings, the only exception being natural causes of death in patients with PD only treated as outpatients only (SMR 1.0, 95% CI 0.8–1.3). In outpatients, all-cause mortality was doubled compared to the general population, with significantly higher SMRs both in PD only (1.3, 95% CI 1.1–1.5), PD & SMI (2.3, 95% CI 1.8–3.0), and PD & SUD (5.3, 95% CI 4.5–6.3). For patients admitted to hospital, SMRs were markedly higher (7.0, 95% CI 6.5–7.6) than in patients treated solely in outpatient clinics.

**Table 2:** All-cause and cause-specific standardized mortality ratios for patients with a personality disorder diagnosis with and without psychiatric comorbidity

	All PD patients			PD only			PD & SMI			PD & SUD^a^		
	Deaths	SMR	95% CI	Deaths	SMR	95% CI	Deaths	SMR	95% CI	Deaths	SMR	95% CI
All causes	890	3.8	(3.6–4.0)	248	2.0	(1.7–2.2)	181	3.7	(3.2–4.3)	461	7.6	(6.9–8.3)
Natural causes	416	2.2	(2.0–2.5)	161	1.6	(1.4–1.9)	94	2.4	(1.9–2.9)	161	3.5	(3.0–4.1)
Cancer	125	1.4	(1.2–1.6)	63	1.3	(1.0–1.6)	29	1.5	(1.0–2.1)	33	1.5	(1.1–2.1)
Cardiovascular	92	2.4	(2.0–2.9)	39	1.9	(1.4–2.6)	27	3.3	(2.2–4.8)	26	2.7	(1.9–4.0)
Respiratory	47	4.0	(3.0–5.3)	13	2.0	(1.2–3.5)	10	3.6	(1.9–6.6)	24	9.3	(6.2–13.8)
Mental disorders	32	7.9	(5.6–11.2)	5	2.3	(1.0–5.6)	3	3.3	(1.1–10.3)	24	24.8	(16.6–37.0)
Other natural	120	3.0	(2.5–3.6)	41	1.9	(1.4–2.6)	25	3.0	(2.0–4.4)	54	5.2	(4.0–6.8)
Unnatural causes	450	11.0	(10.0–12.0)	79	3.8	(3.0–4.7)	81	11.1	(8.9–13.8)	290	22.5	(20.1–25.3)
Poisoning	151	14.0	(11.9–16.4)	11	2.0	(1.1–3.6)	12	6.4	(3.6–11.2)	128	36.7	(30.9–43.7)
Suicide	251	15.0	(13.2–17.0)	61	7.2	(5.6–9.2)	64	21.6	(16.9–27.6)	126	23.9	(20.1–28.5)
Other unnatural	48	3.6	(2.7–4.8)	7	1.0	(0.5–2.2)	5	2.0	(0.9–4.9)	36	8.8	(6.3–12.2)
Missing	24	3.9	(2.6–5.8)	8	2.4	(1.2–4.9)	6	4.9	(2.2–11.0)	10	6.0	(3.2–11.2)
Inpatients												
All causes	558	7.0	(6.5–7.6)	114	5.3	(4.4–6.4)	124	5.2	(4.3–6.2)	320	9.4	(8.4–10.4)
Natural causes	245	3.8	(3.4–4.3)	76	4.3	(3.3–5.3)	62	3.1	(2.4–4.0)	107	4.1	(3.4–4.9)
Unnatural causes	299	23.1	(20.6–25.9)	36	12.4	(8.8–17.2)	59	19.1	(14.5–24.4)	204	29.4	(25.5–33.5)
Outpatients only												
All causes	332	2.1	(1.9–2.4)	134	1.3	(1.1–1.5)	57	2.3	(1.8–3.0)	141	5.3	(4.5–6.3)
Natural causes	171	1.4	(1.2–1.6)	85	1.0	(0.8–1.3)	32	1.6	(1.1–2.3)	54	2.8	(2.1–3.6)
Unnatural causes	151	5.4	(4.6–6.3)	43	2.4	(1.8–3.2)	22	5.2	(3.4–7.9)	86	14.5	(11.8–17.9)

*CI* confidence interval, *Obs* number of deaths, *PD* personality disorder, *SMI* severe mental illness, *SMR* standardized mortality ratio, *SUD* substance use disorder

^a^Includes patients with PD & SUD, with and without SMI

Comparative analyses within the PD group showed that mortality was markedly higher in all age groups for both men and women with comorbid SMI and SUD, compared to those with PD only (Table 3). The HR was particularly high in those with PD & SUD, the most vulnerable groups being women aged 20–34 years (HR = 7.2, 95% CI 4.5–11.7) and men aged 35–49 (HR = 5.7, 95% CI 3.8–8.8).

**Table 3:** Sex- and age stratified hazard ratios for mortality in patients with personality disorder with comorbid severe mental illness and/or substance use disorder, compared to patients with personality disorder only

Sex	Age at entry	PD only				PD & SMI					PD & SUD^a^				
		Patients	Person-years	Deaths	HR	Patients	Person-years	Deaths	HR	95% CI	Patients	Person-years	Deaths	HR	95% CI
Women	All ages	11,000	43,957	138	1.0	3,830	16,185	103	1.9	(1.4–2.4)	4917	20,303	193	3.6	(2.9–4.5)
	20–34	5517	20,674	22	1.0	1797	7490	19	2.4	(1.3–4.5)	2628	10,736	75	7.2	(4.5–11.7)
	35–49	3834	16,327	43	1.0	1342	5904	30	1.9	(1.2–3.2)	1567	6,841	57	3.4	(2.3–5.1)
	50–79	1649	6956	73	1.0	691	2791	54	1.7	(1.1–2.3)	722	2,725	61	2.4	(1.7–3.4)
Men	All ages	6432	24,452	110	1.0	2003	8105	78	1.9	(1.5–2.6)	4703	18,153	268	3.6	(2.9–4.6)
	20–34	2766	9872	22	1.0	769	3080	18	2.4	(1.2–4.2)	2398	9,259	103	4.8	(3.0–7.4)
	35–49	2536	10,202	26	1.0	809	3506	13	1.3	(0.7–2.8)	1685	6,597	102	5.7	(3.8–8.8)
	50–79	1130	4377	62	1.0	425	1520	47	2.0	(1.4–3.0)	620	2,297	63	2.1	(1.5–3.0)

*CI* confidence interval, *HR* hazard ratio, *PD* personality disorder, *SMI* severe mental illness, *SUD* substance use disorder

^a^Includes patients with PD & SUD, with and without SMI

Sensitivity analyses with three different comorbidity groups showed coinciding SMRs between the two groups PD & SUD (but no SMI), and PD & SUD & SMI (Table S1). Sensitivity analyses of individuals with a repeated PD diagnosis showed somewhat lower SMRs due to both natural and unnatural causes of death, in both the PD only and the comorbid groups. The pattern of higher SMRs in the comorbid groups remained, however, consistent (Table S2).

## Discussion

Our study shows that patients with PD have almost four times higher mortality than the general population, with highest SMRs for poisoning and suicide. Relative mortality was substantially higher in patients diagnosed with SMI and/or SUD in addition to PD. The mortality in patients with PD and comorbid SUD compared to the mortality in the general population was extremely high for unnatural causes of deaths. These patients had an almost four times higher all-cause mortality HR than patients with PD only. Poisoning was the cause of death with the overall highest SMRs in patients with PD & SUD group, but the increased SMRs were also present in natural causes of death. The mortality was highest in the inpatient group. For patients with comorbid PD & SMI, the HR was doubled.

The conceptualization of PD in ICD-10 is broader than in the DSM-IV and DSM-5 [4, 56], which may affect the comparison between studies, but still our findings on overall increased mortality is in accordance with findings from previous studies [12, 38–40, 42, 43, 57–60]. In our cohort, F60.3 Emotionally unstable PD was the most frequent subtype (30.3%). This is in line with Kuo and coworkers, where 33.4% had borderline PD [12]. Emotionally unstable PD (ICD-10) or borderline PD (DSM 5) are, however, often more frequent diagnoses in inpatient cohorts, probably due to the severe clinical characteristics and increased need for hospital care in these patients. A possible explanation of the distribution in our study may hence be the inclusion of outpatients, as well as the inclusion of patients treated in somatic health care with a PD diagnosis.

Even so, we find that all-cause, natural, and unnatural SMRs for inpatients in our study are in line with other recent studies [12, 42], but with slightly higher SMR for unnatural causes (23.1, 95% CI 20.6–25.9) than Kuo and coworkers (15.5; 12.3–18.7) [12]. For patients with outpatient care only, we found somewhat lower all-cause SMR (2.1; 1.9–2.4) than Björkenstam and coworkers [43], where total SMRs were 3.7 (3.3–4.1) in women and 3.8 (3.4–4.2) in men.

Our finding of a very high SMRs among patients with comorbid PD & SUD is in line with other studies showing that alcohol and drug use significantly increases mortality in individuals with PD [12, 47, 50, 61]. Crump and coworkers demonstrated that mortality due to accidents, which included death by poisoning, was higher than suicide mortality in all mental disorders, with PD at the highest risk with a four-to-sevenfold increase [46].

The high mortality in individuals with SMI or SUD is well known [36, 40, 60, 62]. The reasons for the increased mortality for patients when PD is comorbid with SMI or SUD are not clear, but severe personality pathology is characterized by lack of emotional regulation and may, as a consequence, lead to aggravation of self-destructive or risk-taking behavior under the influence of substance use; with or without intention [46, 63].

A more fundamental problem of the PD conceptualization is the generally high degree of comorbidity, both between different PDs, but also with other mental disorders, probably caused by personality disturbances having a more dimensional rather than categorical quality. The dimensional classification of PDs in ICD-11 aims at eliminating this “artificial comorbidity”, both for research and clinical purposes [64]. The pattern of high mortality in the comorbid groups in our study may thus reflect a more general symptom severity, with personality disturbances in a complex interplay with other mental symptoms, rather than a pattern of high mortality within distinct categories. It could also be that registered comorbidity with SMI represents diagnostic insecurity rather than co-existing fulfilment of separate diagnostic criteria.

In contrast to the generally higher SMRs in the comorbid groups compared to the PD only group, the SMRs due to natural causes of death in inpatients with PD & SMI or PD & SUD was, somewhat surprisingly, similar or lower than SMR in the PD only group. This finding may simply be a chance finding or it may be due to a more extensive follow-up of somatic conditions in the comorbid groups, or lower degree of help-seeking behavior for somatic conditions in the PD only group.

### Strengths and limitations

The study includes a nationwide, complete data sample from both inpatient and outpatient specialized psychiatric and somatic health care in a country with publicly funded health care services for the entire population, ensuring generalizable estimates of mortality. The validity of clinically based registry diagnoses is often questioned, but high agreement in Norwegian registry and research based diagnoses of SMI has been shown [65] and a recent validity study from the Swedish National Patient Register reports an agreement of 93% for PD diagnoses according to ICD-10 criteria, somewhat lower for DSM criteria [56]. The validity of data on underlying cause of death in the Norwegian CDR is high [66]. In the PD & SUD group in our study, “mental disorders” as an underlying natural cause of death is, however, used more often than in the PD & SMI and PD only group (5%, 1.7% and 2%, respectively). This may indicate a lower validity of registered causes of death in the PD & SUD group.

We included all individuals with at least one PD diagnosis, which may result in a lower diagnostic specificity of PD. Subgroup analyses of the group of individuals with only one registered PD diagnosis showed a higher proportion of men with a slightly higher age (2.6 years older at entry and 4 years older at death), and a higher rate of natural causes of death, in particular alcohol related liver disease (ICD-10 K70). To avoid selection bias through excluding a subgroup of patients with possible personality disturbances and lower rates of help-seeking behavior, we hence included patients with only one registered PD diagnosis, resulting in somewhat higher SMRs, but a similar pattern concerning mortality and comorbidity.

We chose to include individuals up to 79 years, which may have introduced uncertainty with respect to age related disorders, such as neurodegenerative diseases. However, very few studies address PD in older adults, and as underlined in a recent study by Penders and coworkers, there is an urgent need for studies investigating both age adequate assessment and treatment options as well as epidemiological aspects of PD in older age [67]. Additional analyses including individuals up to 69 years only reduced the number of deaths with 8% and the point estimates for SMRs and hazard ratios presented above (Table 3) were only marginally changed (results not shown). The relatively short follow-up represents a limitation that may lead to both under- and overestimation of SMR. There may also be an inflated prevalence of comorbidity in clinical samples (Berkson’s bias[25]), but underreporting of SMI and/or SUD in patients with PD is also possible. In addition, survivor bias could have led to underestimation of SMR in the comorbid groups; as the presence of two diagnoses may be associated with a more extensive follow-up period than one PD diagnosis only. The lack of control of unmeasured mortality risk factors is also an important limitation.

## Conclusions

Our study shows extremely high mortality in patients with PDs and comorbid substance use disorders, or with a comorbid diagnosis of schizophrenia, bipolar disorder or severe depression, compared to the general population. PD patients with comorbid substance use disorders have a fourfold increased risk compared to PD patients without such diagnoses, with young female patients being at highest risk. In patients with comorbid schizophrenia, bipolar disorder or severe depression, the risk is doubled. The mortality is particularly high in patients who were treated as inpatients, but the increased total mortality is also true for patients treated as outpatients only. The most profoundly increased mortality is due to unnatural causes of death, in particular poisoning and suicide. Although strongly associated with comorbidity, the mortality is also high in patients with personality disorders only, compared to the general population. Thus, the higher mortality in patients with personality disorders described in many previous studies cannot fully be accounted for by comorbidity.

### Implications

The results from our study implicate that special emphasis should be placed on patients with PD & SUD comorbidity. These patients may be challenging to treat, as they often show complex symptomatology and reduced ability to develop stable relationships which may hamper treatment alliance. Cooperative strategies should nevertheless be implemented for comorbid patients with PD & SUD treated in both mental health care and drug abuse treatment facilities, to avoid “gaps” in treatment and follow-up. While preventing natural causes of death may be more relevant in patients with comorbid PD & SMI, targeted strategies to avoid premature mortality must be developed to prevent deaths due to unnatural causes in patients with comorbid PD & SUD.

## Supplementary Information

Below is the link to the electronic supplementary material.

Supplementary file1 (DOCX 20 KB)

## Data Availability

The data that support the findings of this study are available from the Norwegian Patient Registry and the Norwegian Cause of Death Registry. Restrictions apply to the availability of these data, which were used under license for this study. For information on how to access the data see https://www.helsedirektoratet.no/tema/statistikk-registre-og-rapporter/helsedata-og-helseregistre/norsk-pasientregister-npr/sok-om-data-fra-npr
