# Supplementary material for: Total and cause-specific mortality in patients with personality disorders: the association between comorbid severe mental illness and substance use disorders
Source: Soc Psychiatry Psychiatr Epidemiol. 2021 Mar 7;56(10):1809–19. doi: 10.1007/s00127-021-02055-3 (PMC8429406; doi:10.1007/s00127-021-02055-3)
Supplement: Supplementary file 1 — Supplementary file1 (DOCX 20 KB) [file 127_2021_2055_MOESM1_ESM.docx]

**Høye A, Jacobsen BK, Bramness JG, Nesvåg R, Reichborn-Kjennerud T, Heiberg I: The impact of psychiatric comorbidity and substance use disorders on total and cause-specific mortality in patients with personality disorders**

**Supplementary tables 1 and 2**

**Table S1**. Standardized mortality ratios for patients with personality disorder diagnosis. Comparison of results in the original model to results in the subgroups PD & SUD without SMI and PD, SUD and SMI combined.

|  | **PD & SUD** | |  |  | **PD & SUD (no SMI)** | | |  | **PD & SUD & SMI** | | |
| --- | --- | --- | --- | --- | --- | --- | --- | --- | --- | --- | --- |
|  | Obs | SMR | 95 % CI |  | Obs | SMR | 95 % CI |  | Obs | SMR | 95 % CI |
| All causes | 461 | 7.6 | (6.9-8.3) |  | 250 | 7.2 | (6.4-8.2) |  | 211 | 8.2 | (7.1-9.3) |
| Natural causes | 161 | 3.5 | (3.0-4.1) |  | 88 | 3.4 | (2.8-4.2) |  | 73 | 3.7 | (2.9-4.6) |
| Unnatural causes | 290 | 22.5 | (20.1-25.3) |  | 157 | 20.1 | (17.2-23.5) |  | 133 | 26.3 | (22.2-31.2) |
| Abbrevations: CI, confidence interval; Obs, number of deaths; PD, personality disorder; SMI, severe mental illness; SMR, standardized mortality ratio; SUD, substance use disorder | | | | | | | | | | | |

**Table S2**. All-cause and cause-specific standardized mortality ratios for patients with personality disorder diagnosis. Comparison of results in the original model to results in the subgroup with a repeated PD diagnosis.

|  |  | **All PD patients** | | |  | **PD only** | | |  | **PD & SMI** | | |  | **PD & SUD** | | |
| --- | --- | --- | --- | --- | --- | --- | --- | --- | --- | --- | --- | --- | --- | --- | --- | --- |
|  | | Obs | SMR | 95% CI |  | Obs | SMR | 95% CI |  | OBs | SMR | 95% CI |  | Obs | SMR | 95% CI |
| Original model | |  |  |  |  |  |  |  |  |  |  |  |  |  |  |  |
|  | All causes | 890 | 3.8 | (3.6-4.0) |  | 248 | 2.0 | (1.7-2.2) |  | 181 | 3.7 | (3.2-4.3) |  | 461 | 7.6 | (6.9-8.3) |
|  | Natural causes | 416 | 2.2 | (2.0-2.5) |  | 161 | 1.6 | (1.4-1.9) |  | 94 | 2.4 | (1.9-2.9) |  | 161 | 3.5 | (3.0-4.1) |
|  | Unnatural causes | 450 | 11.0 | (10.0-12.0) |  | 79 | 3.8 | (3.0-4.7) |  | 81 | 11.1 | (8.9-13.8) |  | 290 | 22.5 | (20.1-25.3) |
|  |  |  |  |  |  |  |  |  |  |  |  |  |  |  |  |  |
| Only one recorded PD diagnosis | | | |  |  |  |  |  |  |  |  |  |  |  |  |  |
|  | All causes | 249 | 5.1 | (4.5-5.7) |  | 68 | 2.8 | (2.2-3.5) |  | 55 | 5.2 | (4.0-6.7) |  | 126 | 9.0 | (7.6-10.7) |
|  | Natural causes | 128 | 3.2 | (2.7-3.8) |  | 49 | 2.4 | (1.8-3.2) |  | 30 | 3.3 | (2.3-4.7) |  | 49 | 4.5 | (3.4-6.0) |
|  | Unnatural causes | 116 | 15.3 | (12.8-18.4) |  | 18 | 4.9 | (3.1-7.8) |  | 24 | 19.1 | (12.8-28.5) |  | 74 | 27.9 | (22.2-35.0) |
|  |  |  |  |  |  |  |  |  |  |  |  |  |  |  |  |  |
| At least 2 PD diagnoses | |  |  |  |  |  |  |  |  |  |  |  |  |  |  |  |
|  | All causes | 641 | 3.5 | (3.2-3.7) |  | 180 | 1.8 | (1.5-2.1) |  | 126 | 3.3 | (2.8-3.9) |  | 335 | 7.2 | (6.5-8.0) |
|  | Natural causes | 288 | 2.0 | (1.8-2.2) |  | 112 | 1.4 | (1.2-1.7) |  | 64 | 2.1 | (1.6-2.7) |  | 112 | 3.2 | (2.7-3.9) |
|  | Unnatural causes | 334 | 10.0 | (9.0-11.1) |  | 61 | 3.5 | (2.8-4.6) |  | 57 | 9.4 | (7.3-12.2) |  | 216 | 21.1 | (18.5-24.1) |
| Abbrevations: CI, confidence interval; Obs, number of deaths; PD, personality disorder; SMI, severe mental illness; SMR, standardized mortality ratio; SUD, substance use disorder | | | | | | | | | | | | | | | | |
